# Acute Coronary Syndrome Treatment Costs from the Perspective of the Supplementary Health System

**DOI:** 10.5935/abc.20150129

**Published:** 2015-10

**Authors:** Vanessa Teich, Tony Piha, Lucas Fahham, Haline Bianca Squiassi, Everton de Matos Paloni, Paulo Miranda, Denizar Vianna Araújo

**Affiliations:** 1 MedInsight, São Paulo, SP – Brazil; 2 AstraZeneca Brasil, Cotia, SP – Brazil; 3 Orizon, São Paulo, SP – Brazil; 4 Departamento de Clínica Médica da Universidade do Estado do Rio de Janeiro, Rio de Janeiro, RJ – Brazil

**Keywords:** Acute Coronary Syndrome / economy, Health Care Costs, Health Expenditures, Data Interpretation, Statistical, Prepaid Health Plans

## Abstract

**Background:**

Acute coronary syndrome (ACS) is defined as a “group of clinical symptoms compatible with acute myocardial ischemia”, representing the leading cause of death worldwide, with a high clinical and financial impact. In this sense, the development of economic studies assessing the costs related to the treatment of ACS should be considered.

**Objective:**

To evaluate costs and length of hospital stay between groups of patients treated for ACS undergoing angioplasty with or without stent implantation (stent+ / stent-), coronary artery bypass surgery (CABG) and treated only clinically (Clinical) from the perspective of the Brazilian Supplementary Health System (SHS).

**Methods:**

A retrospective analysis of medical claims of beneficiaries of health plans was performed considering hospitalization costs and length of hospital stay for management of patients undergoing different types of treatment for ACS, between Jan/2010 and Jun/2012.

**Results:**

The average costs per patient were R$ 18,261.77, R$ 30,611.07, R$ 37,454.94 and R$ 40,883.37 in the following groups: Clinical, stent-, stent+ and CABG, respectively. The average costs per day of hospitalization were R$ 1,987.03, R$ 4,024.72, R$ 6,033.40 and R$ 2,663.82, respectively. The average results for length of stay were 9.19 days, 7.61 days, 6.19 days and 15.20 days in these same groups. The differences were significant between all groups except Clinical and stent- and between stent + and CABG groups for cost analysis.

**Conclusion:**

Hospitalization costs of SCA are high in the Brazilian SHS, being significantly higher when interventional procedures are required.

## Introduction

Acute coronary syndrome (ACS) is defined by the American Heart Association as a “group of clinical symptoms compatible with acute myocardial ischemia”. Its clinical spectrum includes unstable angina and acute myocardial infarction (AMI) with or without ST-segment elevation.

According to Polanczyk and Ribeiro^1^, prevalence data in Brazil estimate that 5% to 8% of adults older than 40 years old have ACS^1^. The disease is the leading cause of mortality in Brazil^2^ and developed countries^3^. It is estimated that for every 5 to 7 cases of myocardial infarction there is one death^4,5^. Thus, coronary heart disease is the leading cause of death worldwide, making it one of the diseases with the highest clinical and financial impact^4^.

Several types of interventions have been shown to be beneficial for the management of ACS, including the use of medications such as antiplatelet agents, beta-blockers, heparin, glycoprotein IIb/IIIa inhibitors and the use of procedures such as catheterization and thrombolytic therapy such as coronary angioplasty and revascularization^6^.

Currently in Brazil there are no studies comparing the costs of different types of treatment for ACS in SHS. Studies such as this are needed to make it possible to evaluate the economic impact a disease such as ACS has on society.

Thus, the objective of this article is to evaluate the costs and the length of hospital stay between groups of patients that were treated for ACS, submitted to angioplasty with or without stenting (stent + / stent-), revascularization (CABG) and treated only clinically (Clinical), from the perspective of the Brazilian Supplementary Health System (SHS).

## Methods

A retrospective analysis was carried out of medical claims from beneficiaries of health care provided by private institutions in all Brazilian regions (excluding the states of Tocantins, Roraima and Mato Grosso do Sul), through a database obtained from Orizon, a health care company responsible for the management of information processes from 110 health insurance companies, representing more than 18 million beneficiaries in Brazil. This database included data from patients undergoing hospital treatment for ACS and costs related to hospitalization by type of procedure (food, exams, medical gases, hygiene/cosmetics, fees, materials, drugs, procedures and taxes) and length of hospital stay. The period considered for the analysis was between January 2010 and June 2012.

Orizon carried out the preliminary analysis of the data and MedInsight performed the statistical analysis. The treatments included in the analysis for the ACS episode management were: medical treatment, angioplasty with stenting, angioplasty without stenting (balloon angioplasty) and coronary artery bypass grafting (CABG).

Quantitative variables such as cost and length of stay were described by the mean, median and mode. An exploratory analysis through Q-Q Plots method was performed to define the normality of the extracted data, and the Shapiro-Wilk normality test was applied to determine the adherence of the sample to a normal distribution. In cases of non-normal distributions, the nonparametric Kruskal-Wallis test was applied, used to determine equality between groups, and the Nemenyi-Damico-Wolfe-Dunn post-hoc Test, to test the difference between groups after the Kruskal-Wallis test. Analyses were performed using the R Statistical Software, version 3.1.1^7^. A significance level of 5% was used.

## Results

A total of 2,876 patients were identified in the period between 1/2010 and 6/2012, being divided into four groups: patients treated by angioplasty with stenting (*stent*+) patients treated by angioplasty without stenting (*stent*-) patients undergoing revascularization (CABG) and patients treated clinically (Clinical), all of them using antiplatelet agents. The mean age of patients in each group ranged between 55 and 65 years (55 years in the Clinical group, 59 years in the CABG group, 62 years in the stent+ group and 65 years in the stent- group), whereas the percentage of female patients ranged from 18% to 24% (22% in the Clinical group, 20% in the CABG group, 24% in the stent+ group and 18% in the stent- group; p = 0.51).

Patient characteristics were similar between groups, with significant difference in the mean age between the Clinical group and patients from groups submitted to angioplasty with or without stent (Clinical *vs* stent-, p = 0.003; Clinical *vs* stent*+*, p = 0.016).

After the sample selection, total hospital costs for the same period (between 1/2010 and 6/2012) were extracted and divided by procedure, as shown in Table 1.

**Table 1:** Total cost of hospital treatment by type of cost

Type of cost	Clinical	stent-	stent+	CABG
Food	R$ 8,735.28	R$ 62,470.20	R$ 205,822.95	R$ 39,885.35
Examination	R$ 122,649.76	R$ 559,699.66	R$ 4,374,932.76	R$ 574,814.36
Medical Gases	R$ 72,369.09	R$ 198,039.56	R$ 848,664.42	R$ 217,487.94
Hygiene/Cosmetics	R$ 897,14	R$ 1,125.15	R$ 10,062.67	R$ 1,823.80
Fees	R$ 62,786.31	R$ 131,953.21	R$ 1,425,054.16	R$ 276,275.32
Materials	R$ 196,965.06	R$ 2,139,035.25	R$ 55,820,543.70	R$ 2,629,796.09
Medications	R$ 357,560.41	R$ 961,490.33	R$ 4,855,775.35	R$ 971,924.45
Procedures	R$ 16,969.35	R$ 1,101,482.66	R$ 13,435,554.79	R$ 2,219,036.26
Taxes	R$ 311,558.95	R$ 1,210,965.32	R$ 8,724,484.12	R$ 1,654,359.39
Others	R$ 0,00	R$ 841,11	R$ 3.696,23	R$ 104,40
Total	R$ 1,150,491.35	R$ 6,367,102.45	R$89,704,591.15	R$ 8,585,507.36

CABG: Coronary artery bypass surgery.

The analysis of total costs showed that the highest costs in the Clinical group were related to medications, followed by fees, materials and exams. In the stent- group, higher costs were associated with the use of materials, followed by fees, procedures and use of medications. In the stent+ group, the higher costs were related to the use of materials, followed by procedures, fees and medications. Finally, in the CABG group, the higher costs were associated with the use of materials, followed by procedures, fees and medications. The results of the analysis of the mean costs per procedure, segmented by group, are shown in Table 2.

**Table 2:** Mean cost per procedure by type of cost

Type of cost	Clinical	stent-	stent+	CABG
Food	R$ 138.66	R$ 300.34	R$ 85.69	R$ 188.14
Examination	R$ 1,946.82	R$ 2,690.86	R$ 1,821.37	R$ 2,711.39
Medical Gases	R$ 1,148.72	R$ 952.11	R$ 353.32	R$ 1,025.89
Hygiene/Cosmetics	R$ 14.24	R$ 5.41	R$ 4.19	R$ 8.60
Fees	R$ 996.61	R$ 634.39	R$ 593.28	R$ 1,303.19
Materials	R$ 3,126.43	R$ 10,283.82	R$ 23,239.19	R$ 12,404.70
Medications	R$ 5,675.56	R$ 4,622.55	R$ 2,021.56	R$ 4,584.55
Procedures	R$ 269.35	R$ 5,295.59	R$ 5,593.49	R$ 10,467.15
Taxes	R$ 4,945.38	R$ 5,821.95	R$ 3,632.17	R$ 7,803.58
Others	R$ 0.00	R$ 4.05	R$ 1.53	R$ 0.49
Total	R$ 18,261.77	R$ 30,611.07	R$ 37,345.79	R$ 40,497.68

CABG: Coronary artery bypass surgery.

The median costs among the four groups were compared using the Kruskal-Wallis method, which showed a p-value < 0.001, rejecting the hypothesis of equality between the costs. A post‑hoc test was used to perform the pairwise comparison, as shown in Table 3.

**Table 3:** Cost comparison between groups

	Clinical	stent+	stent-	CABG
Clinical		S	NS	S
stent+			S	NS
stent-				S
CABG				

S: Significant; NS: Non-significant; CABG: Coronary artery bypass surgery.

The comparison analysis of the median costs of treatment, in the period between 1/2010 and 6/2012, indicated that the difference was not significant when comparing the Clinical group with stent- group and in the comparison between the *stent*+ group and CABG group. All other comparisons showed statistically significant differences.

**Figure 1:**
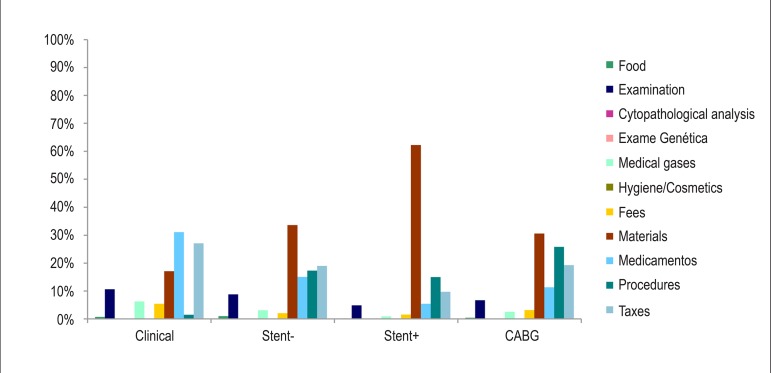
Percentage of average costs by type of cost and analyzed group; CABG: Coronary artery bypass surgery.

The representativeness of the types of cost in the four analyzed groups is shown in Figure 1.

The chart analysis shows that the stent-, stent+ and CABG groups had higher cost with materials and procedures (representing > 50% of the total cost of each group), while in the Clinical group this cost is only 18%. The Clinical group showed that most of the costs are related to medications and fees (58%), which was expected, as the cost is basically restricted to the use of medications and consultations.

The results of the analysis of hospital stay of the four groups and the mean cost per day of hospitalization are shown in Table 4.

**Table 4:** Mean length of stay and mean cost of hospitalization

Admission	Clinical	stent-	stent+	CABG
Mean (DP)	9.19 days (6,7)	7.61 days (8.1)	619 days* (12)	15.20 days* (7.3)
Median	8 days	6 days	5 days	14 days
Mode	5 days	2 days	2 days	14 days
Mean cost - Admission day	R$ 1,987.03	R$ 4,024.72	R$ 6,033.40	R$ 2,663.82

* Significant difference compared to the Clinical group; CABG: Coronary artery bypass surgery.

Patients in the Clinical group showed a minimum hospital stay of two days and a maximum of 35 days. In the stent+ patients group, the hospital stay varied from one day to a maximum of 515 days. Patients in the stent- group had a maximum length of stay of 80 days, while in the CABG group patients showed a variation in hospital stay from four to 50 days.

To test the normality of the data related to the length of stay, exploratory analysis was performed through a QQ Plot graphic, and non-adherence to a normal distribution was confirmed by the Shapiro-Wilk test (p < 0.001). Therefore, it was decided to analyze the data by non-parametric methods. Thus, when comparing the mean length of hospital stay, the mean costs among the four groups were compared using the Kruskal‑Wallis method, which showed a p-value < 0.001, rejecting the hypothesis of equality between lengths of hospitalization. A post-hoc test was used to perform the pairwise comparison, as shown in Table 5.

**Table 5:** Length of stay comparison between groups

	Clinical	stent+	stent-	CABG
Clinical		S	NS	S
stent+			S	S
stent-				S
CABG				

S: significant; NS: non-significant; CABG: Coronary artery bypass surgery.

**Figure 2:**
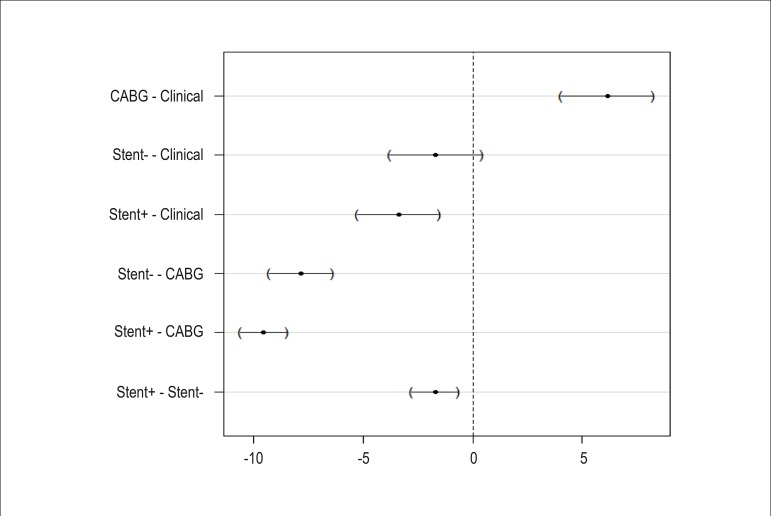
Mean length of stay difference and 95%CI; CABG: coronary artery bypass surgery.

Regarding the median hospitalization time, only the comparison of the Clinical group *versus* the stent- group was not significant. All other comparisons showed significant results. These results can be confirmed graphically in Figure 2, where the confidence interval of the difference between mean lengths of hospitalization crosses the vertical axis of the graph only for the comparison between Clinical and stent- groups.

## Discussion

An analysis was performed of the data related to medical claims of Supplementary Health System patients with ACS, clinically treated without intervention, patients undergoing angioplasty with or without stenting and patients undergoing CABG. The patients that were only clinically treated were considered the control group in this analysis.

An important finding of this analysis is related to the fact that the mean cost results did not show a statistically significant difference between the clinically treated group and the group submitted to angioplasty without stenting, as well as between the group treated by CABG and the group submitted to angioplasty with stenting. This finding suggests that patients treated with angioplasty without stenting and those submitted only to clinical treatment have similar treatment costs, which can be explained by the lower complexity of angioplasty, often performed on an outpatient basis and with shorter hospital length of stay.

Patients undergoing CABG and those submitted to angioplasty with stent implantation showed similar costs between them and higher costs when compared to less complex procedures (angioplasty without stent and clinical treatment), representing significant expenditures for the treatment of patients with ACS.

A retrospective study carried out in France, involving 154 patients with ACS and submitted to angioplasty with stent implantation in 2005, concluded that the costs involved in performing this procedure have a financial impact for hospitals^8^.

Another study carried out in Brazil measured direct and indirect costs related to the treatment of ACS, from the perspectives of the Unified Health System (SUS) and Supplementary Health System. The study considered the historical series of hospitalizations in SUS between 1999 and 2010 and the expected number of hospitalizations for 2011 projected by a linear extrapolation of the historical series and concluded that the estimated direct cost associated with ACS in 2011, from the SUS perspective, is approximately 0.77% of the total SUS budget, and from the SHS perspective, this estimate would come to R$ 515.138.617^9^.

Studies like this demonstrate the importance of following these patients, the pharmacological treatment and lifestyle changes that can contribute to preserving the health of patients and prevention of complications, in order to prevent patients from undergoing complex treatments that may excessively burden the health care system.

A limitation of the present study is the lack of a reliable national registry of cases of cardiovascular diseases and hence, the scarcity of supplementary medical data and other health care providers, as this study used data from health insurance companies linked to the Orizon^©^ company.

## Conclusions

In the present study it was observed that the clinical treatment and angioplasty without stenting procedure, associated with the use of antiplatelet agents, are less onerous for the SHS compared to major procedures such as angioplasty with stenting and CABG, as, due to the high degree of complexity, these procedures had higher associated costs and therefore should be considered as relevant costs to the health system.
